# The surgical management of scoliosis: a scoping review of the literature

**DOI:** 10.1186/s13013-014-0026-3

**Published:** 2015-01-09

**Authors:** Nathan Evaniew, Tahira Devji, Brian Drew, Devin Peterson, Michelle Ghert, Mohit Bhandari

**Affiliations:** Division of Orthopaedics, Department of Surgery, McMaster University, 293 Wellington St N, Suite 110, Hamilton, ON L8L 8E7 Canada; Department of Clinical Epidemiology and Biostatistics, McMaster University, Hamilton, ON Canada

**Keywords:** Scoliosis, Spinal deformity, Scoping review, Systematic review, Clinical epidemiology

## Abstract

**Background:**

Scoping reviews are innovative studies that can map a range of evidence to convey the breadth and depth of a large field. An evidence-based approach to the wide spectrum of surgical interventions for scoliosis is paramount to enhance clinical outcomes. The objectives of this scoping review were to identify critical knowledge gaps and direct future research.

**Methods:**

This study was completed according to the methodology of Arksey and O’Malley. Two reviewers performed duplicate systematic screening of eligibility. Studies were classified according to patient age, scoliosis etiology, outcomes reported, study design, and overall research theme.

**Results:**

There were 1763 eligible studies published between 1966 and 2013. The literature focused on adolescents (83% of studies) with idiopathic scoliosis (72%). There was a dominance of observational designs (88%), and a paucity of randomized trials (4%) or systematic reviews (1%). Fifty six percent of studies were conducted in North America, followed by 23% in Europe and 18% in Asia. Few high-level studies investigated surgical indications, surgical approaches, surgical techniques, or implant selection. Patient important outcomes including function, health-related quality of life, pain, and rates or re-operation were infrequently reported.

**Conclusions:**

Current research priorities are to (1) undertake high-quality knowledge synthesis and knowledge translation activities; (2) conduct a series of planning meetings to engage clinicians, patients, and methodologists; and (3) clarify outcome reporting and strategies for methodological improvement. Higher-quality studies are specifically needed to inform surgical indications, surgical approaches, surgical techniques, and implant selection. Engaging global partners may increase generalizability.

## Introduction

‘Scoliosis’ encompasses a heterogeneous group of coronal and rotational spinal deformities that can affect patients of any age. Adolescent idiopathic scoliosis alone is associated with a substantial burden of health care utilization, but costs are even higher for patients with congenital or neuromuscular etiologies [1-5]. Likewise, degenerative scoliosis may affect up to 68% of adults greater than 70 years old and is a frequent cause of pain and disability [6,7]. An evidence-based approach to the wide range of surgical interventions for scoliosis is paramount to enhance clinical outcomes.

Knowledge translation is the dynamic and iterative process of summarizing, disseminating, exchanging, and applying research findings to improve patient outcomes and strengthen health care systems [8]. Comprehensive systematic reviews are the foundation of most knowledge translation activities, but understanding very broad or complex topics can be challenging. Systematic reviews related to the surgical management of scoliosis have been limited by narrow scope, heterogeneity across the included studies, or insufficient primary evidence [2,5,9,10].

Scoping reviews are innovative studies that can map a range of evidence to convey the breadth and depth of a large field. Scoping reviews are also powerful tools to guide ongoing knowledge synthesis and inform future research [11]. In contrast to standard systematic reviews, scoping reviews ask broader questions and do not perform detailed assessments of individual studies. Scoping reviews also differ from narrative reviews in that they comprehensively and reproducibly identify relevant articles in order to minimize bias. Arksey and O’Malley’s six-stage framework, which involves a systematic literature search, duplicate screening of eligibility, and the identification of overall research themes, is the foundation of modern scoping review methodology [11,12].

This study is a scoping review that was performed to synthesize the available literature reporting on the surgical management of scoliosis. The objectives of this study were to (1) identify critical knowledge gaps and (2) direct future research.

## Methods

### Eligibility criteria

All therapeutic clinical studies examining the surgical management of scoliosis were included. No restrictions were placed for patient age, scoliosis etiology, or date of publication. Studies of only non-surgical interventions and non-therapeutic study designs such as economic, prognostic, and diagnostic studies were excluded. Non-clinical research studies such as cadaveric biomechanical studies and basic science studies were excluded. Conference proceedings describing unpublished studies and studies that were published in languages other than English or could not be retrieved in English full-text were excluded. Narrative reviews and case reports of less than 5 patients were counted but excluded from the analysis.

### Identification of studies

MeSH and EMTREE headings and subheadings were used in various combinations to query MEDLINE and EMBASE (up to June 6, 2013) in Ovid for potentially eligible articles (ie. “scoliosis/su [surgery] AND surgical procedures, operative/or orthopedics/su or spinal fusion/or general surgery/”). The headings were supplemented with free text to increase sensitivity (ie. “[scoliosis.ti,ab. OR curv*.ti,ab.] AND [operation or operative or operate or surgery or surgical).ti,ab.]”). The search strategy was also adapted in PubMed (up to June 6, 2013) to search for articles e-published ahead of print and not yet indexed on Ovid.

### Screening and data extraction

Two reviewers performed duplicate screening of all titles and abstracts for eligibility using a piloted electronic screening form (Distiller SR, Evidence Partners 2013, Ottawa ON, Canada). All discrepancies were resolved through consensus.

Patient age and scoliosis etiology were classified according to the recommendations of the Scoliosis Research Society Terminology Committee and Working Group [13]. Reported outcomes were classified as radiological, functional, pain, rates of reoperations, rates of complications, physical exam outcomes, laboratory results, operative variables (such as blood loss or operating time), or other. All applicable classifications were recorded for each study. Total sample size, year of publication, and primary country of were also collected. The geographical distribution of studies was not adjusted for population or research density within each continent.

### Study designs and levels of evidence

The two reviewers independently assessed study designs using the Centre for Evidence-Based Medicine in Oxford guidelines for therapeutic studies [14,15]. Higher quality randomized controlled trials (RCTs) were classified as Level I, while lesser quality RCTs and prospective non-randomized controlled studies were classified as level II. Retrospective controlled studies were classified as Level III, and uncontrolled studies were classified as Level IV. Reviewers were not blinded to authors, publication information, or any published level of evidence descriptions [16].

### Literature themes

The two reviewers compiled a set of potential primary study themes through discussion and consensus after completing title and abstract screening [11]. The two reviewers then piloted the themes for face validity and content validity using a sample of 50 included studies. Minor revisions were made to clarify existing themes, add additional themes, and document discriminatory criteria for each theme. The single most relevant primary theme for each included study was collected, recognizing that some secondary themes would not be captured.

‘Levels’ described studies that reported on the selection of spinal levels for fusion; ‘Approaches and Stages’ described studies that reported on the effects of varying surgical approaches, adjunctive peri-operative interventions, or timing of consecutive procedures; ‘Implants and Techniques’ described studies that reported on the use of specific implant systems or varying surgical techniques related to implants. ‘Indications’ described studies that reported on the effect of an intervention in a specific or varying set of populations; ‘Grafts’ described studies that reported on the effect of varying graft materials; ‘Blood’ described studies that reported on interventions to minimize blood loss; ‘Infection’ described studies that reported on interventions to prevent or treat infections; ‘Anaesthesia’ described studies that reported on anaesthetic agents or techniques; ‘Neuromonitoring’ described studies that reported on neuromonitoring procedures and techniques; ‘Analgesia’ described studies that reported on methods to treat post-operative pain; ‘Rehabilitation’ described studies that reported on interventions related to rehabilitation in operatively treated patients; ‘Psychological’ described studies that reported on interventions to improve psychological outcomes.

### Analysis

Inter-observer agreement for the reviewers’ assessments of study eligibility was calculated with Cohen’s kappa coefficient of agreement [17]. Inter-observer agreement for the reviewers’ assessments of levels of evidence was calculated with the Intraclass Correlation Coefficient (IBM SPSS Version 21; Chicago IL, 2012). Descriptive statistics were used to summarize all other data. Discrete variables are reported as counts or proportions, normally distributed continuous variables are summarized as means with standard deviations, and skewed continuous variables are summarized as medians with interquartile ranges.

## Results

### Search results

**Figure 1:**
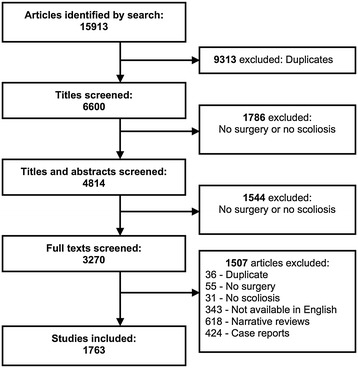
**Flow diagram depicting the screening and review of potentially eligible articles.**

The search strategy identified 15913 potentially relevant articles (Figure 1). Of these, 9313 were removed because they were duplicate references to the same articles from multiple databases. A further 1786 were excluded during screening of titles and 1544 were excluded during screening of titles and abstracts because they either did not relate to surgery or they did not relate to scoliosis. Of 3270 articles eligible for full text review, 618 were excluded because they were narrative reviews, 424 were excluded because they were case reports, 343 were excluded because they were not available as full-texts in English, and 122 were excluded because they were not relevant or were duplicates. In total, 1763 studies were included for data extraction and further analysis. Agreement between the two reviewers for eligibility was satisfactory (kappa = 0.78).

### Characteristics of included studies

**Figure 2:**
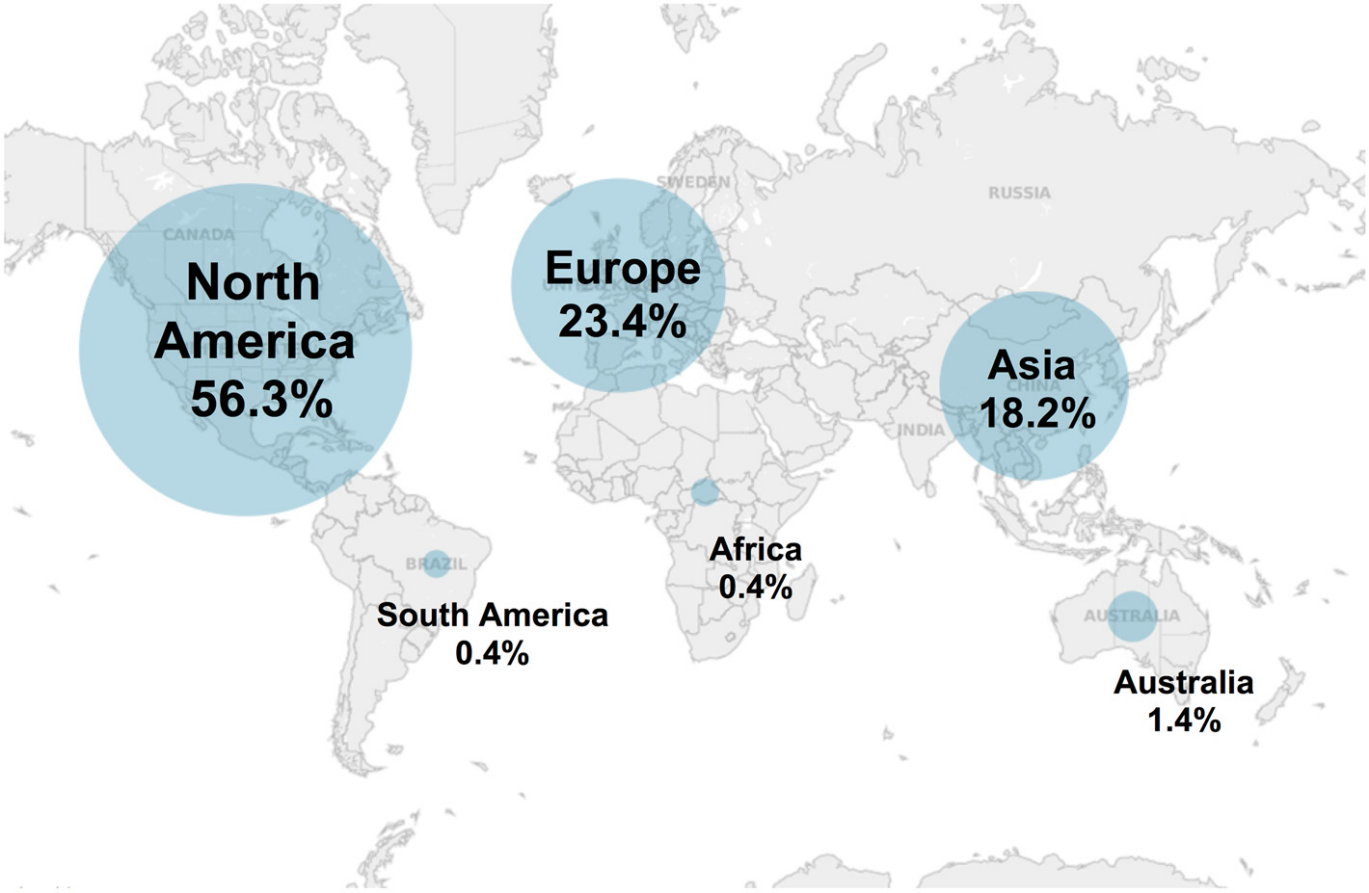
**Global distribution of clinical research reporting on the surgical management of scoliosis.** Percentages reflect raw proportions and are not adjusted for population or researcher density.

**Figure 3:**
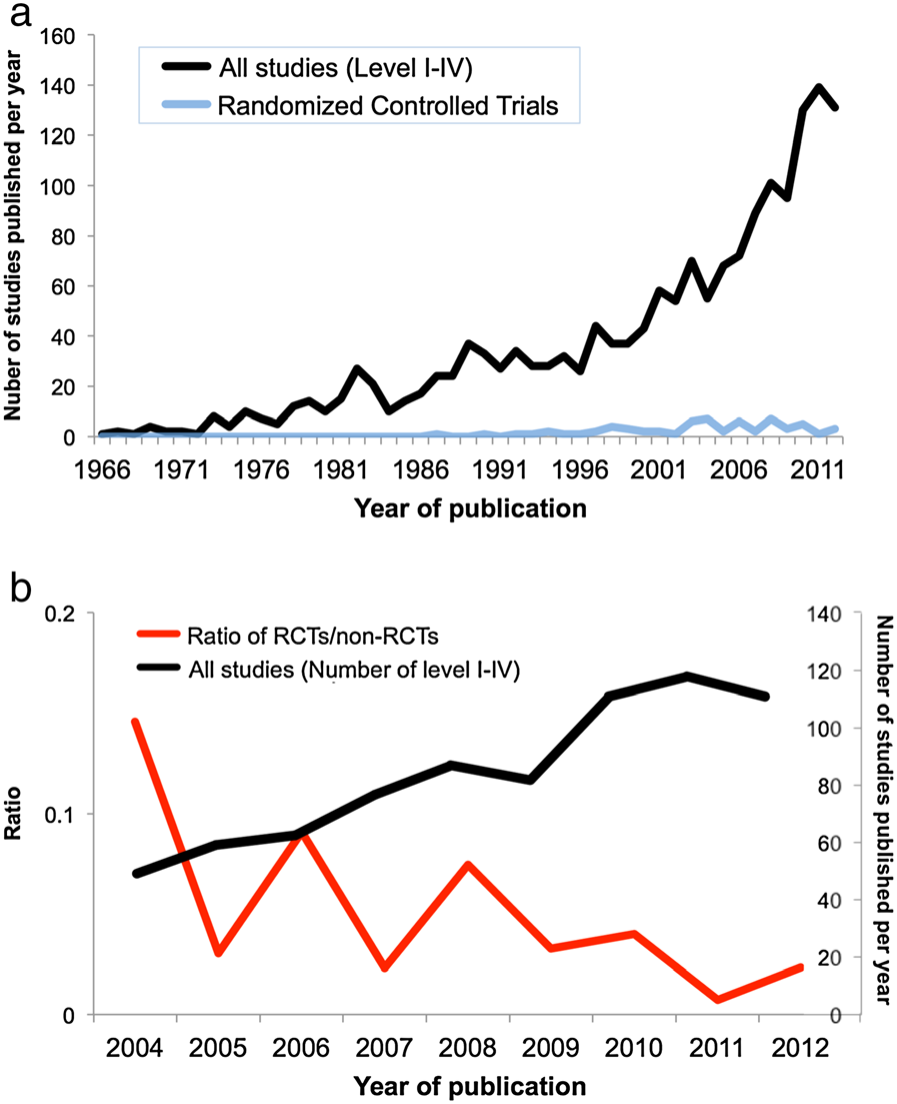
**Temporal distribution of clinical research reporting on the surgical management of scoliosis. (a)** Total volume of clinical research reporting on the surgical management of scoliosis over time; **(b)** Ratio of randomized controlled trials (RCTs) to non-RCTs since 2004 superimposed against the total volume of clinical research.

Overall, 993 (56%) of the studies were conducted in North America, followed by 413 (23%) in Europe and 320 (18%) in Asia (Figure 2). Twenty-three studies were conducted by Australia and New Zealand together, and only seven each were conducted in each of South America and Africa. The total number of identified studies published globally per year rose from just one in 1966 to more than 130 in each of 2010, 2011, and 2012 (Figure 3a). Studies were most frequently published in *Spine* (711 studies), *European Spine Journal* (167), *Journal of Pediatric Orthopaedics* (142), *Journal of Bone and Joint Surgery - American Volume* (99), and *Clinical Orthopaedics and Related Research* (75).

**Figure 4:**
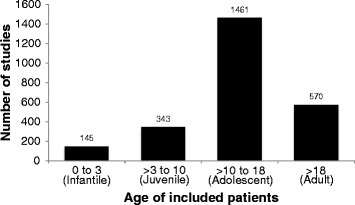
**Distribution of clinical research reporting on the surgical management of scoliosis by age of included patients.** All applicable age categories were recorded for each study.

**Figure 5:**
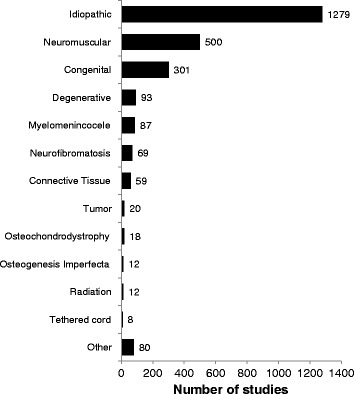
**Distribution of clinical research reporting on the surgical management of scoliosis by etiology.** All applicable etiological categories were recorded for each study.

**Figure 6:**
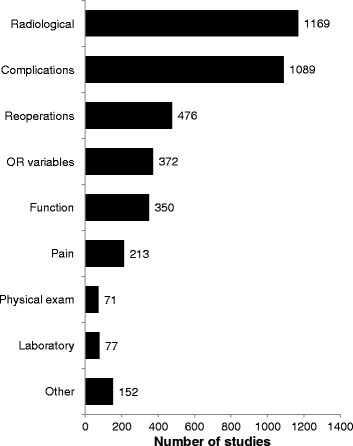
**Distribution of clinical research reporting on the surgical management of scoliosis by reported outcomes.** All applicable outcomes categories were recorded for each study.

The most frequently included age category was adolescent (83% of studies) (Figure 4), and the most frequently included etiology of scoliosis was idiopathic (72%) (Figure 5). Patients with neuromuscular scoliosis were included in 28% of studies and patients with congenital scoliosis were included in 17% of studies. Despite being a frequent cause of pain and disability in older adults, patients with degenerative scoliosis were included in just 5% of the identified studies [6,7]. More than one age category of patients was applicable in 33% of studies, and more than one etiological classification of scoliosis was applicable in 23% of studies. Radiological outcomes were reported in 66% of studies, rates of complications were reported in 62% of studies, and rates of reoperations were reported in 27% of studies (Figure 6). Functional outcomes or health-related quality of life were reported in just 20% of studies. The median sample size across all studies was 42 (IQR 24 to 87).

### Study designs and levels of evidence

**Figure 7:**
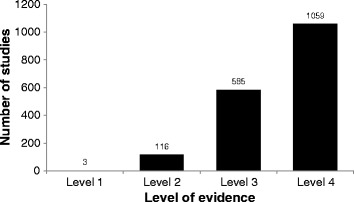
**Distribution of clinical research reporting on the surgical management of scoliosis by level of evidence.**

There were 65 prospective randomized controlled trials (4% of the included studies), 115 (7%) prospective non-randomized controlled studies, 571 (32%) retrospective controlled studies, and 983 (56%) uncontrolled studies (case series). Despite a dramatic increase in the total number of studies over time, the proportion of studies that were randomized controlled trials remained low, and has actually relatively decreased following a peak in 2004 (Figure 3b). There were 15 systematic reviews (<1%) and 14 systematic reviews and meta-analyses (<1%). Only three studies were classified as level I (<1%) and only 116 were graded as level II (7%), while 585 were classified as level III (33%) and 1059 were classified as level IV (60%) (Figure 7). Agreement between the two reviewers for levels of evidence was satisfactory (ICC = 0.771).

### Literature themes

**Figure 8:**
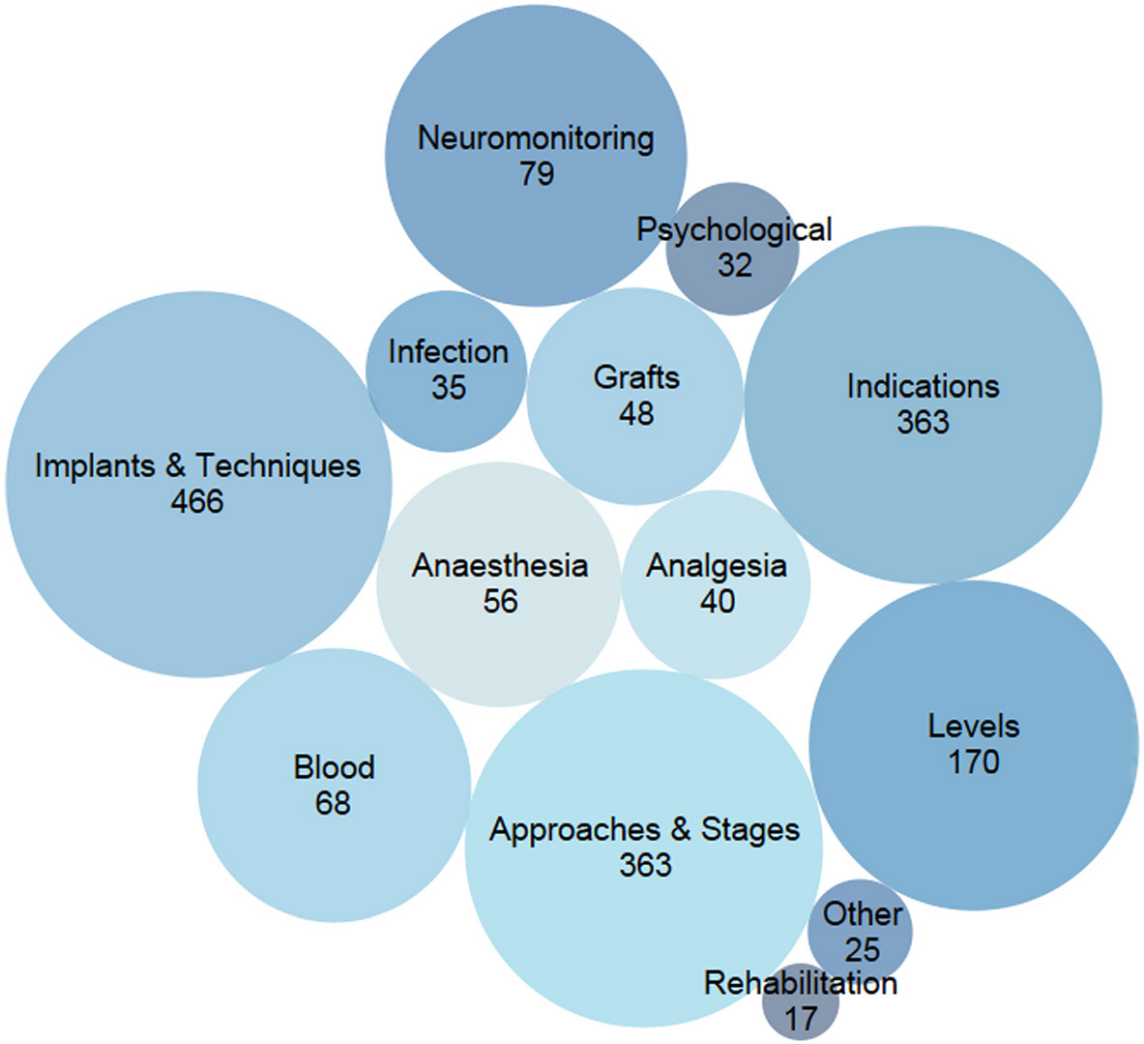
**Illustrative plot of the primary research themes across studies reporting on the surgical management of scoliosis.** The single most relevant primary theme was selected for each study. The size of each circle is proportional to the number of studies for each primary theme. The circle locations and colors are arbitrary.

Studies most frequently investigated the effects of specific implants and specific surgical techniques (26%), followed by approaches and staging (21%), and indications for surgery (21%) (Figure 8). Ten percent of studies investigated the selection of spinal levels for fusion, 5% investigated neuromonitoring, and 4% investigated strategies to manage blood loss. Three percent investigated anaesthesic management, 3% investigated bone grafts or the use of bone graft substitutes, and 2% investigated post-operative pain management. Only 35 studies (2%) investigated the prevention or management of surgical site infections, 32 (2%) investigated interventions to improve psychological outcomes, and 17 (1%) investigated post-operative rehabilitation.

Of the 65 identified RCTs, 55 related to perioperative surgical care rather than direct surgical considerations: 19 investigated anesthetic management, 13 investigated strategies to manage blood loss, 13 investigated post-operative pain management, and 5 investigated neuromonitoring. Four RCTs investigated surgical approaches or staging, 4 investigated bone grafts, 3 investigated implants or techniques, and 1 investigated the selection of spinal levels for fusion. Overall, 61 of the 65 RCTs reported on adolescent patients, and 60 reported on idiopathic curves. The median sample size of the RCTs was 36 (IQR 30 to 50), and the global distribution of RCTs paralleled the overall global distribution shown in Figure 2. Of the 14 meta-analyses, three each investigated indications for surgery, approaches and staging, implants and techniques, and psychological outcomes, and one each investigated blood loss and post-operative pain management.

## Discussion

This study was a scoping review performed to summarize the literature available to guide the surgical management of scoliosis, identify critical gaps in current knowledge, and direct future research. The majority of the identified literature focused on adolescent patients with idiopathic scoliosis. There was a clear dominance of uncontrolled studies, and a striking paucity of RCTs. Few high-level studies investigated surgical indications, surgical approaches, surgical techniques, or implant selection. Patient important outcomes including function, health-related quality of life, pain, and rates or re-operation were infrequently reported.

### Limitations

Of the 3270 studies identified for full-text screening, 343 full-texts could not be retrieved in English. Retrieving and translating non-English studies for systematic reviews can be technically prohibitive, but excluding them may produce misleading or exaggerated findings, particularly when estimating the global distribution of research outside of North America and Europe [18]. Fortunately, large RCTs are most often widely available in high-impact English language journals, and the 343 excluded studies represent only approximately 10 percent of the eligible sample of studies. In their study of 130 systematic reviews, Moher et al. established that language restrictions in systematic reviews of conventional interventions do not seem to produce meaningful bias [18]. The relative lack of studies from India and China may reflect a tendency to publish in journals not indexed in the search databases or it may reflect a developing research infrastructure [19]. This issue highlights an opportunity to engage global partners in future studies [20].

The thematic framework was developed *ad hoc* and the identified domains have not been previously reported. This study’s application of the scoping review framework to the scoliosis literature was entirely novel, and the thematic domains were developed after reviewing all titles and abstracts according to the Arksey and O’Malley framework [11,12]. Themes, age, etiology, and reported outcomes were not extracted in duplicate, but consensus meetings were used to document clear definitions and ensure consistency. Further, the themes were piloted by each of the reviewers for face- and content- validity.

### Implications for research

Critical knowledge gaps and directions for future research are summarized in Table 1. The first priority is to focus on knowledge synthesis and effective knowledge translation in order to optimize the impact of existing research. This scoping review identified 1763 relevant articles; however, even with such a large number of publications, there were only 15 prior systematic reviews and 14 prior meta-analyses. This scoping review can guide a series of high-quality focused systematic reviews on clinically important topics with identified robust data. Likewise, this scoping review can also inform evidence-based decision aids or preliminary clinical practice guidelines [11].

**Table 1:** **Research gaps and future research directions for the surgical management of scoliosis**

**Research gaps**	**Future research directions**
There are few focused systematic reviews relative to the extensive scoliosis literature, reflecting a lack of emphasis on knowledge synthesis and knowledge translation.	**Knowledge synthesis:** Perform a series of high-quality focused systematic reviews examining important clinical questions.
There is a striking paucity of randomized controlled trials (RCTs), and the existing RCTs are characterized by generally small sample sizes.	**Knowledge translation:** Use existing systematic reviews to inform a series of evidence-based decision aids and preliminary clinical practice guidelines.
Very few high-level studies have investigated surgical indications, surgical approaches, surgical techniques, or implant selection.	**Future RCTs:** Conduct a series of surveys or planning meetings to engage clinicians, patients, methodologists, and other knowledge users in the design and conduct of future large RCTs.
Patient important outcomes such as function, health-related quality of life, pain, and rates or re-operation have been infrequently reported in comparison to radiological outcomes and rates of complications.	**Ongoing scoping work:** Clarify inconsistent outcome reporting and identifying practical strategies for methodological improvement.

The second priority is to engage clinicians, patients, methodologists, and other knowledge users in the design and conduct of future large RCTs. A series of planning meetings could clarify research questions, strengthen a collaborative network, and optimize strategies for successful potential funding applications. There were only 65 RCTs identified, and these trials were generally characterized by small sample sizes. Less than one quarter of these trials addressed primarily surgical research questions such as surgical indications, approaches, techniques, or implant selection. Adequately powered large RCTs of surgical interventions are challenging to conduct, but multiple trials over the last decade have demonstrated their feasibility and potential clinical impact [21,22], and the scoliosis literature already contains many examples of multi-center collaborations [23].

The final priority is to clarify inconsistent outcome reporting and identify practical strategies for methodological improvement. Radiological outcomes are critical to understand deformity correction and technical success, but it remains unclear whether particular radiological outcomes are used consistently in the literature. In addition, it is apparent that radiological outcomes may not always correlate with patient reported function, quality of life, or body image [24-26]. Observational designs dominate the scoliosis literature, but they are frequently prone to confounding bias, selection bias, transfer bias, and recall bias [27]. Further research is necessary to investigate whether methodological safeguards can minimize tendencies towards exaggerated or misleading results [28].

## Conclusions

There exists a broad and varied body of research to guide the surgical management of scoliosis. Current research priorities are to (1) undertake high-quality knowledge synthesis and knowledge translation activities; (2) conduct a series of planning meetings to engage clinicians, patients, and methodologists; and (3) clarify outcome reporting and strategies for methodological improvement. Higher-quality studies are specifically necessary to evaluate surgical indications, surgical approaches, surgical techniques, and implant selection. Future studies may also consider engaging global partners to increase generalizability.
